# A global inventory of animal diversity measured in different grazing treatments

**DOI:** 10.1038/s41597-022-01326-1

**Published:** 2022-05-16

**Authors:** Tianna Barber-Cross, Alessandro Filazzola, Charlotte Brown, Margarete A. Dettlaff, Amgaa Batbaatar, Jessica S. J. Grenke, Isaac Peetoom Heida, James F. Cahill

**Affiliations:** 1 Department of Biological Sciences, University of Alberta, Edmonton, AB T6G 2E9 Canada; 2 Department of Biological Sciences, University of Toronto Scarborough, 1265 Military Trail, Toronto, Canada; 3 Desert Laboratory on Tumamoc Hill, University of Arizona, Tucson, AZ 85745 USA; 4 Department of Agricultural, Food and Nutritional Science, University of Alberta, Edmonton, Alberta T6G 2P5 Canada; 5 Faculty of Land and Food Systems, University of British Columbia, Vancouver, BC V6T 1Z4 Canada

**Keywords:** Conservation biology, Community ecology, Grassland ecology, Agroecology

## Abstract

Grazing by wild and domesticated grazers occurs within many terrestrial ecosystems worldwide, with positive and negative impacts on biodiversity. Management of grazed lands in support of biological conservation could benefit from a compiled dataset of animal biodiversity within and adjacent to grazed sites. In this database, we have assembled data from the peer-reviewed literature that included all forms of grazing, co-occurring species, and site information. We reviewed 3,489 published articles and found 245 studies in 41 countries that surveyed animal biodiversity co-occurring with grazers. We extracted 16,105 observations of animal surveys for over 1,200 species in all terrestrial ecosystems and on all continents except Antarctica. We then compiled 28 different grazing variables that focus on management systems, assemblages of grazer species, ecosystem characteristics, and survey type. Our database provides the most comprehensive summary of animal biodiversity patterns that co-occur with wild and domesticated grazers. This database could be used in future conservation initiatives and grazing management to enhance the prolonged maintenance of ecosystems and ecosystem services.

## Abstract (summary)

**Table:** 

Measurement(s)	Kingdom • Phylum • Class • Order • Taxon • Family • Genus • Species • Year of observation • Survey time period • Grazing estimate • Grazing species • Grazer domestication • Last grazing event • Latitude • Longitude • Elevation • Country • Number of sites • Survey technique • Survey type • Presence of wild grazers • Presence of domesticated grazers • Plant community • Ecosystem class • Fence presence • Tilled status • Herbivores present • Fertilization • Fire history • Land ownership • Year of study initiation • Year of study finished
Technology Type(s)	Data extraction
Sample Characteristic - Organism	Vertebrate • Invertebrate • Plants • Fungi
Sample Characteristic - Environment	Grassland ecosystem • Forest ecosystem • Shrubland ecosystem • Wetland ecosystem • Desert ecosystem • Tundra ecosystem • Coastal ecosystem
Sample Characteristic - Location	Argentina • Australia • Austria • Canada • China • Kingdom of Denmark • England • Ethiopia • Finland • French Republic • Germany • Hong Kong • Hungary • India • Iran • Ireland • Israel • Italy • Kazakhstan • Kenya • Lesotho • Mongolia • Kingdom of the Netherlands • New Zealand • Kingdom of Norway • Poland • Portuguese Republic • Romania • Scotland • Senegal • Republic of South Africa • Kingdom of Spain • Sweden • Switzerland • Tanzania • Tunisia • Uganda • United Kingdom • United States of America

## Background & Summary

Grazing by domesticated livestock is a dominant land-use practice of many ecosystems worldwide, with rangelands occupying 54% of terrestrial land cover^1^. Domesticated livestock grazing is also increasing worldwide, with an expected 30–50% increase in the spatial extent, specifically in countries with high biodiversity^2^. Although not all meat consumption is dependent on grazing, most domesticated livestock managed in rangelands is destined for meat consumption and contributes to the global average of 80 g of meat-based protein consumed daily per person^3^. In recent decades, meat protein has also been increasingly changing from poultry to be derived from cattle, as beef has become more popular and accessible in southeastern Asia, Mexico, and Brazil^4^, leading to a shift in the most common meat animals across the globe. Quantifying the effects of domesticated livestock grazing on natural systems, particularly in comparison to wild grazers that often share the same lands, is thus critical as grazing patterns shift globally. Grazing animals, both wild and domesticated, can have diverse impacts on ecological processes^5–9^. For example, grazing by domesticated livestock can contribute to carbon and nitrogen losses^8^, alter diversity or densities of co-occurring herbivores, pollinators, and detritivores^5,9^, and impact soil microbial communities^7^. A necessary first step to managing diversity and developing sustainable grazing practices is to use a data-focused approach that inventories biodiversity on grazed lands.

One aspect of global concern is grazing impacts on animal biodiversity through the potentially cascading effects of vegetation removal. Previous meta-analyses have independently synthesized the effects of grazing on soil biota^7^, soil characteristics^10^, nutrient cycling^8^, plant communities^11,12^, and animals^9,13,14^. Briefly, terrestrial animals are strongly affected by the presence of large herbivore grazers because of indirect mortality, increased disturbance, and competition for shared vegetation resources potentially resulting in trophic cascades^13–15^. For instance, a meta-analysis on cattle and sheep grazing effects found grazer exclusion resulted in the increased diversity of pollinators and herbivores, decreased detritivore diversity, and inconsistent effects on predator diversity^9^. Despite these recent syntheses of grazer effects on biodiversity, key knowledge gaps remain around management, including differences among ecosystems, grazer species, climate, and intensity^15^. Thus, there is a need for a database that inventories animal diversity patterns associated with domesticated and wild grazers to increase accessibility in exploring these evolving research questions.

Grazing can have effects that are species specific^12,16,17^, vary with management style^8,18^, or are specific to certain habitats. For example, some studies encourage grazing as a conservation strategy to suppress the dominance of invasive or non-native species^17^, but others show the opposite effect with grazing promoting non-native species^16^. Similarly, grazing practices, such as rotational vs. continuous grazing, are also debated in the response of ecosystems^18–20^. Evaluating the effect of domesticated and wild grazing also requires considering the ecological context and survey methodology. For example, grazing in deserts, grasslands, and forests may have different impacts on the ecosystem. In some ecosystems, grazing can suppress grassland invasion into shrublands^19^ and, in others, can promote shrub encroachment into grasslands^20^. The lack of a synthesized database on grazing patterns globally hinders our ability to make well-informed decisions on conservation initiatives for grazed lands in support of biodiversity. Thus, we have created a database to include all forms of grazing management described in the literature and include species-specific information about the target species.

Using a systematic literature review, we aimed to create a global inventory of animal diversity on lands with domesticated or wild grazers. To do so, we reviewed 3,489 published articles and collated data from 245 studies found in 41 different countries. We compiled 16,105 unique observations for over 1,200 animal species. From the papers that included those observations, we also extracted 28 other grazing variables that focus on management systems, assemblages of grazer species, ecosystem characteristics, and survey type. We also included biodiversity data on ungrazed sites if included in the study. This database is a novel and substantial improvement over the data presented in Filazzola *et al*.^9^ by including over 11,000 more observations and 12 new variables that describe the characteristics of grazers and the study area. This database can provide a valuable resource for biological conservation or grazing management towards the maintenance of ecosystems.

## Methods

### Synthesis and data extraction

Data were collected using a literature search of Web of Science for peer-reviewed journal articles published between 1970 and November 2019. We conducted two sets of searches to capture grazing with discrete comparisons (e.g., grazed/ungrazed, moderate vs. heavy intensity grazing) and a range of grazing intensities. The search terms used for each were as follows 1) (graz* OR livestock) AND (exclosure* OR exclusion OR exclude* OR ungrazed OR retire* OR fallow* OR fence* OR paddock*), 2) (“grazing intensity” OR “grazing gradient” OR “stocking rate” OR “rotation*grazing”). Our synthesis includes domesticated and wild grazer species, with the latter defined as an undomesticated species naturally occurring in the study area during the study. Wild grazers are typically native species to the region (e.g., the American bison in Western North America) but can include non-native species that are naturalized in the area (e.g., feral horses on Sable Island).

We excluded any study that did not test the effect of grazing animals. A grazer was defined using the definition provided by the authors of the respective study to account for the proportion of forage types in a herbivore’s diet that varies between seasons and habitats. For example, we included animals where their diet is assumed to come from all (e.g., cattle, sheep), most (e.g., wapiti, kangaroos), or some (e.g., deer species) grass species. However, within the included studies, these animals were classified as grazers as most of their diet was grass for the duration of the study. For added clarity about the herbivore composition in each study, we extracted a list of any herbivores listed in the paper regardless of foraging type or if any data was provided.

We only included studies that measured animal diversity or abundance as a response variable and included data we could extract or contact the author to obtain^9^. We included any study with a grazing treatment and included observations within these studies of any grazed and ungrazed sites. All studies with grazing included a comparison to either ungrazed sites, different grazing practices (e.g., cattle vs. sheep), and/or differences in intensities (e.g., heavy/light, extensive/intensive). Studies that only measured plants or soil biota were excluded because syntheses of grazing effects on these groups have already been conducted^7,11,12^, and our goal was to provide a robust inventory of animal diversity. However, if a study included plants, lichens, or fungi in addition to animals, we included this data. Studies discussing marine grazing or aquatic systems were also excluded. From these preliminary filters, we identified 3,489 published manuscripts. We reviewed these 3,489 published articles and found 245 studies that surveyed animals in grazed sites. In total, we extracted 16,105 observations for over 1,200 species.

We extracted 28 variables that focus on management systems, assemblages of grazer species, ecosystem characteristics, and survey type (Table 1). The latitudes, longitudes, and elevations of each study were included when provided for use with geospatial data. In addition, we included variables about the study site’s disturbance history, including last time grazed, if a flood event or fire had occurred, if fertilization was used, if the area was open or fenced off, and if the area was publicly or privately owned. Furthermore, the timeline for the study (i.e., the years the authors initiated and completed the study) was also provided. Study initiation was described by the authors and could include when the grazing treatment started, another treatment was applied, and/or animal surveys began. These timeline columns can be useful in identifying long-term studies and differentiating single grazing events or multi-year experiments. Finally, we generalized the characteristics of the ecosystem of the sites used in each study based on the climate and dominant vegetation.

**Table 1:** The attributes and description of the *metadata.csv* file that lists the general characteristics of each study.

Attribute (column header)	Description of attribute
UniqueID	A unique identifier given to each study and is found in all datasheets.
Title	The title of the study the data was extracted from.
Authors	The authors of the study the data was extracted from.
Source title	The title of the source the study was published in (e.g., journal name).
Publication year	The year the study was published.
GrazerSpecies	The main domesticated grazer species studied in the publication.
GrazerStatus	The domestication status of the grazer species identified (domesticated, wild, or both).
LastGrazingEvent	The number of years since the ungrazed site comparison (if available) was last grazed.
StudyDuration	The duration of the study in years as described by the authors. One year is the minimum length reported.
Latitude	Latitude of the sites if they were given (decimal degrees). Multiple sites are separated by a semi-colon.
Longitude	Longitude of the sites if they were given (decimal degrees). Multiple sites are separated by a semi-colon.
Elevation	Elevation of the sites if they were given (meters). Multiple sites are separated by a semi-colon.
Country	The country where the study was conducted.
NSites	The number of sites surveyed within the study.
SurveyTechnique	The survey method for collecting the responding animals.
SurveyType	Categories of the general survey methods used in the study. Categories are based off the SurveyTechnique column and simplified based on criteria from *surveyCategories.csv*. Multiple survey types within the same study are separated by a semi-colon.
WildGrazerSpecies	Presence of wild grazers in the study area (yes or no).
DomesticatedGrazerSpecies	Presence of domesticated grazers in the study area (yes or no).
PlantCommunity	The status of the plant community. Either: 1) P = planted/seeded or 2) SA = allowed to self-assemble.
EcosystemClass	The type of ecosystem where the experiment was conducted (e.g., forest, desert, alpine). Multiple locations are separated by a semi-colon.
Fenced	Whether the larger study area (excluding exclosures) was open or fenced.
Tilled	If the study area was tilled or not tilled.
Herbivores	A list of all the herbivores present in the study area mentioned in the paper separated by a semi-colon (not including species lists, such as from appendices).
Fertilization	Fertilizer added within the study sites (yes or no).
Fire	Whether fire was a treatment within the study. Fire mainly included prescribed burns, but natural fire occurred.
Ownership	Whether the study area was publicly or privately owned.
YearInitiated	The year that the study was initiated (although data might not have been collected until later years).
YearFinished	The year that the study ended and the final data collection was obtained.

Within the grazing data, we included information about the grazer when provided, including any measurement of the intensity of grazing (e.g., animals per hectare, the height of residual vegetation). We also provided two columns that detailed whether the study tested grazing effects using a discrete comparison or gradient of intensities (Table 2). The value for the target specimens extracted may represent either a single observation or a summarized statistic (e.g., mean animals per site). We identify unique observations as “count” and summarized statistics by the metric used, such as mean, median, standard deviation (column *stat* in *grazingData.csv*). When possible, we also included any record of other grazers that co-occurred with the observed grazer species. The data for these variables were extracted from the papers by a single researcher who read through each paper and filled in available data on the mentioned variables.

**Table 2:** The attributes and description of the grazingData.csv file that has the extracted data from each study.

Attribute (column header)	Description of attribute
UniqueID	A unique identifier given to each study that exists in all datasheets.
HigherTaxon	Whether the responding species or specimen was a vertebrate, invertebrate, fungus, lichen, or plant.
Kingdom	The kingdom that the target species or group of species belongs to within the study.
Phylum	The phylum that the target species or group of species belongs to within the study.
Class	The class that the target species or group of species belongs to within the study.
Order	The order that the target species or group of species belongs to within the study.
Family	The family that the target species or group of species belongs to within the study.
Genus	Taxonomic genus of measured species or specimen.
Species	Species of measured organisms.
SiteID	Identifier given within the paper for a specific site studied.
Year	The year the specific observation was surveyed. In instances where the observation was an average of multiple years, the year is either separated by a semi-colon for distinct years or a dash to indicate a range of years.
Timeframe	Additional notes about the survey time period, such as month or season, were included in this column.
Replicate	The number of replicates associated with the value calculated within a treatment or control. Left blank if there was a single observation, which would present a single count of an organism.
GrazingCompare	Determination of the grazing contrast. Based on a gradient of intensity (e.g., stocking rate) or a contrast between ordinal groups such as grazed vs. ungrazed.
GrazingLevel	Grazing categories or comparisons for ordinal contrasts. Only applicable for the ordinal/binary compare.
GrazingEstimate	The estimate of grazing used in the study (e.g., number of animals per hectare).
GrazingValue	The value of the grazing estimate used in the study.
Estimate	The response variable used to measure the study species (e.g., abundance, diversity, behavioural, fitness).
Statistic	The statistic used to summarize the response of the target specimen (e.g., mean, median, standard error). If listed as “count”, this represents the plot-level measurement of individuals.
Value	The measured value of the responding specimen.

We extracted information about the target specimen, site, year, experimental replicate, and response estimate (Table 2). We included multiple categorizations of the target species to assist future users in synthesizing similar taxa (Table 2). When a species name or genus was provided, we conducted a search query (see *detailedTaxa.r*) through the global biodiversity information facility (GBIF.org) to determine the taxonomic classification of the species, including kingdom, phylum, order, class, and family. When a species name was not included, we provided the lowest taxonomic resolution available. We also included a broader classification of ‘higherTaxon’ to distinguish plants, fungi, vertebrates, and invertebrates. These columns may help group similar species together for community-level analyses. Lastly, we included the characteristic of the plant community (i.e., planted or self-assembled, tilled, and its vegetation class) when plant data was reported.

### Patterns among studies

**Fig. 1:**
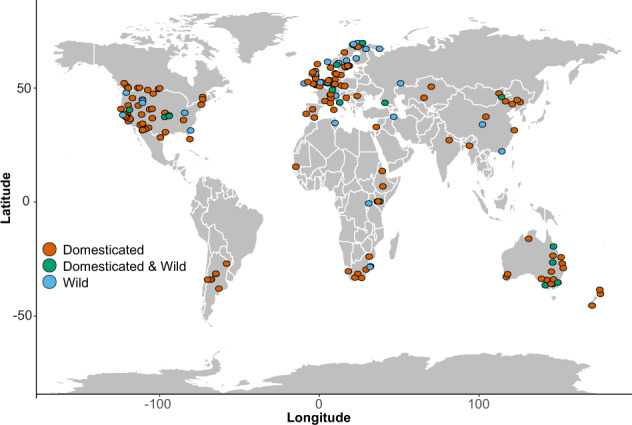
The locations of studies that measured the response of animals to domestic or wild grazing.

**Fig. 2:**
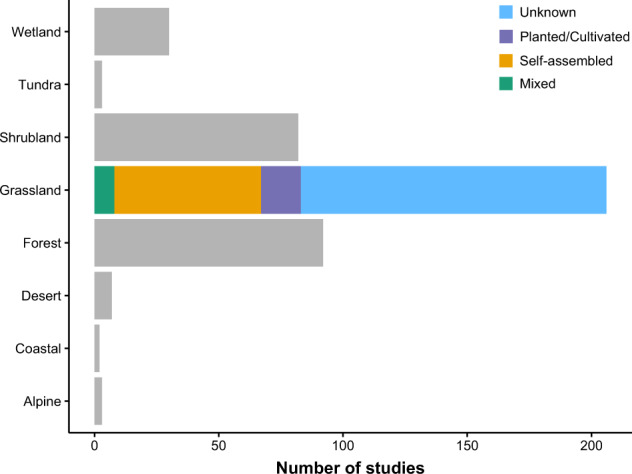
The number of grazing studies conducted in ecosystems around the world. We generalized the characteristics of the ecosystem of the sites used in each study based on the climate and dominant vegetation community. We separated grassland communities into those that were (**a**) semi-natural without recent cultivation or seeding (self-assembled), (**b**) recently cultivated or had supplemental seeding (planted/cultivated), and (**c**) a combination of both. In most grasslands, the cultivation history was unclear.

**Fig. 3:**
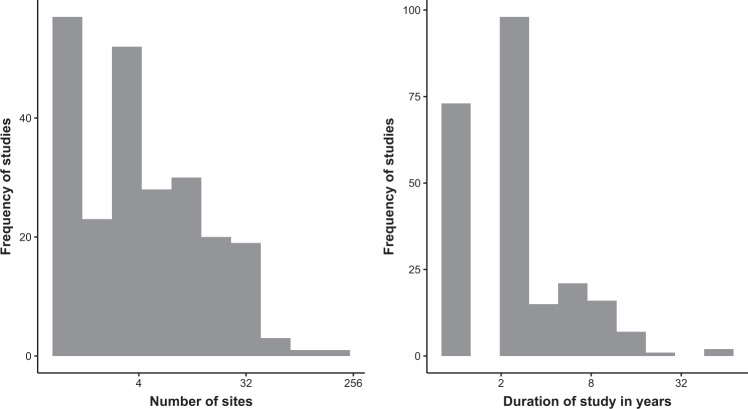
The number of independent sites surveyed and the duration of each study. Most studies were conducted at either a single site or with some replication (e.g., 6–8 sites). Similarly, most studies were either conducted in one year (>30%) or over a few years (e.g., 3–6 years). Very few studies (<5%) used a higher number of sites (>32) or lasted longer than 15 years.

Most of the studies took place in the United States (26%), Australia (9%), and the United Kingdom (7%) (Fig. 1). As expected, most studies were conducted in grasslands (n = 206), followed by forests (n = 92) and shrublands (n = 82) (Fig. 2). We included publications from the entire range of years (i.e., 1970–2019), but most were published after 2000 (76%). The number of sites in a study and the study duration showed a bimodal distribution with a long tail (Fig. 3). Most studies included one to eight different sites, and few were conducted longer than five years (Fig. 3). A few studies were highly replicated, while many were limited in their replication (Fig. 3).

Site and management data were not reported in all studies, as found in other reviews of grazing impacts on ecological processes^10^. Of the studies that mentioned the ownership status of the land used, 46% were on private land, 42% were on public land, and 12% had a history of both public and private ownership. Most studies included binary comparisons (56%) of grazed vs. ungrazed plots or sites, though some also included a discrete (22%) or a continuous estimate of grazing intensities (18%).

**Fig. 4:**
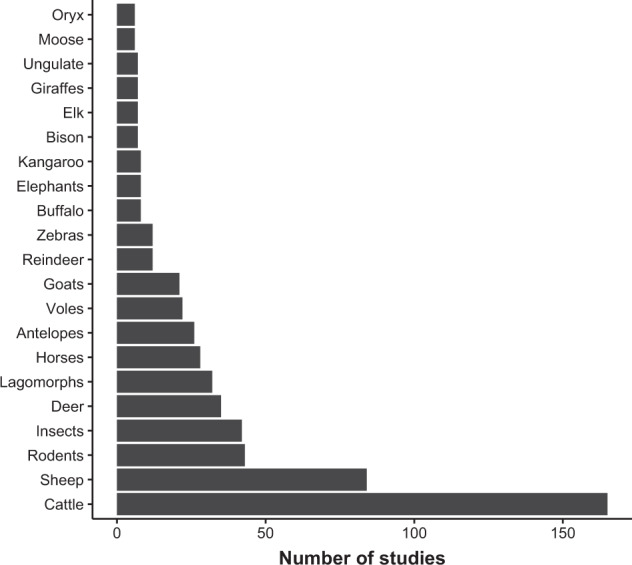
The frequency in which a study reported herbivores. We included any mention of herbivores regardless of being a grazer, browser, granivore, or other class. This list was obtained by the text within the manuscript and is different than the representation of species in the database (i.e., the measured species).

Of the studies that reported plant community origin, 76% were self-assembled, 17% were planted communities, and the remaining included sites were a combination of the two. Domesticated grazers as the focal herbivore made up 67% of the studies, with 12% of the studies having wild grazers as the focal herbivore, and 21% having both present. Domesticated livestock were the most frequently surveyed grazers including cattle (n = 164), sheep (n = 83), and horses (n = 21), but studies are included that examined wild grazers, such as kangaroos (n = 6), elephants (n = 5), and pronghorn (n = 5) (Fig. 4).

## Data Records

The final database consisted of 16,105 unique observations from over 1,200 unique species and at least 221 unique study sites. There are three datasheets: the metadata, grazing data, and study data. The metadata sheet (*metaData.csv*) contains column names for both the grazing and study data, with explanations of each column name (Table 1). The study data (*studyData.csv*) contains the study names, authors, and other identifying information that can narrow down and/or exclude studies that do not meet the researcher’s desired criteria. This includes the year of publication, study duration, focal grazer species, vegetation class, and more. From there, the unique ID codes from the study datasheet can be used to sort and exclude other studies within the grazing datasheet. The grazing datasheet (*grazingData.csv*) contains the data extracted from each paper. The data are categorized by the focal taxa, whether the study was a binary comparison or gradient analysis, and the type of estimate measured. The mean and standard deviation of the estimate is given for each site. The data can be found at The Knowledge Network for Biocomplexity (KNB)^21^.

## Technical Validation

Quality assurance and quality control (QA/QC) was conducted on the entire database of animal responses in grazing sites. We used three steps to evaluate the accuracy of the database, including 1) importation, 2) consolidation, and 3) validation.

### Importation

Separate individuals extracted data from each study and inputted it into a standardized template. One individual was responsible for compiling all the datasets into a master database. Using a primary individual to consolidate the data reduced potential disparities in formatting and allowed a second review of each dataset for transcription errors. Once the database was built, we involved a researcher not involved with the initial extraction to review a subset of the data extracted from each individual. We checked and removed any duplicates that were present in the data. All columns were checked for missing data, incorrect/invalid characters, and data that may have been transcribed into the wrong location. Some data were missing in studies or not available from the authors, such as geospatial coordinates, the species name of the target organism, or a measure of grazing intensity. However, we ensured that data were present for the following columns: *UniqueID, Higher taxon, Taxa, Grazing comparison, Grazing level, Estimate, Statistic*, and *Value* (Table 2). These values are crucial for any meta-analytic techniques to test grazing effects on animal diversity using these data. After compilation, we reviewed these columns for completeness and revisited any missing information. The replicate column was also managed to ensure completeness but is missing values when the measure of animal diversity was at the lowest survey level (e.g., plot, transect, or trap level).

### Consolidation

We examined the data for similarities between studies. In OpenRefine 3.3, we identified alternate representations of the same data using a nearest neighbour analysis of string characters based on Levenshtein distance^22^. This analysis solved two inconsistencies within the database. Firstly, it removed incorrect characters, misspelled words, or differences in case. Secondly, we determined similarly described variables that could be combined into a single definition, which increased consistency among studies. For instance, two studies may have reported units in “biomass (kg)” and “biomass (kilograms)”, which are the same unit and thus can be consolidated. We applied this methodology to all columns with string characters within *grazingData.csv*. A list of all the consolidated or corrected variables can be found in the JSON files within the database. In instances where there were no obvious groupings, we have provided files that suggest biodiversity measures and grazing intensity estimates that could be analogous across studies (*estimateCategories.csv* and *grazingCategories.csv*).

### Validation

We reviewed all values within the database to ensure each was transcribed correctly. All study locations were mapped to examine any apparent inconsistencies with the coordinates, such as observations in the ocean (Fig. 1). We examined histograms and density plots of the values extracted from the dataset to determine if entry errors could explain any outliers. Outliers were reviewed and corrected if necessary. The number of sites and duration of studies had a bimodal distribution (Fig. 3) that could be explained by most studies being narrow in either space (few sites) or time (single year).

The values extracted from each study ranged from 0 to 151,356, where the latter was a study that used biomass in micrograms. Similarly, the number of replicates used in each study ranged from 1 to 400. To help identify if data was incorrectly extracted from manuscripts, we calculated the log-transformed ratio of means (function *escalc*, package *metafor*^23^) and flagged any study where there was an anomalous mean difference (LRR > 5.0) or high pooled variance (variance > 10). We reviewed any flagged observations with the original study to ensure accuracy.

## Usage Notes

A subset of the database presented in this manuscript was used in a previous meta-analysis by Filazzola *et al*.^9^. In that study, the authors explored the effects of sheep and cattle grazing on animal diversity in binary comparisons (i.e., grazed vs. ungrazed). A more versatile database would include inventories of animals on all types of grazed sites (e.g., intensive vs. extensive, wild vs. domesticated), does not require an ungrazed site for comparison, and includes a detailed description of grazing practices and land characteristics. The database presented here contains substantially more information than the synthesis provided by Filazzola *et al*.^9^ in several ways. Firstly, this database includes additional variables, including 12 new columns in the *studyData.csv* and six new columns in the *grazingData.csv*. These columns include more detailed descriptions about the study locations (e.g., fire history, land practices), grazing practices, and the taxonomy of the target species. Secondly, our database includes more observations from additional studies that a) tested grazers other than sheep or cattle, such as wild grazers, and b) included comparisons other than binary grazed vs. ungrazed. The revised database includes many different types of grazing comparisons based on varying levels of intensity, frequency, and grazing practices. These observations represented over 11,000 rows and a 200% increase from the meta-analysis by Filazzola *et al*.^9^. This expanded database could answer new questions such as the importance of grazing relative to other disturbance practices (e.g., fire, fencing) or the impact of grazing at different intensities and frequencies. Finally, our database presented here involved rigorous quality control and quality assurance. This included all the methods described in the Technical Validation section above and an expansion of data to include more specificity. For example, Filazzola *et al*.^9^ reported the centroid latitude and longitude for studies, whereas we included the latitude and longitude for every unique site within a study when reported. The enhanced spatial resolution could allow for pairing with rasterized data such as downscaled climate projections, soil characteristic maps, or topographical information. We also substantially improved the target species’ taxonomic classification, providing the entire taxonomic rank to the lowest resolution available. We believe the presented database will undoubtedly offer great utility relative to the data in Filazzola *et al*.^9^ for users interested in understanding the effects of grazing on animal diversity.

In addition to the consolidation completed above, we provide a list of further refinements that could be applied to group certain studies together. For example, similar measures of animal abundance such as biomass, density, and the number of individuals are not interchangeable but are related. For this reason, we include subjective categorizations of the estimate and grazer estimate columns to assist future users interested in examining similarities among studies. These are available in separate data files (*estimateCategories.csv* and *grazingCategories.csv*).

We encourage future users of this database to take advantage of the site and duration columns provided, which could be a proxy for replication across space and time, respectively. We have also provided a complete list of herbivores that the authors mentioned in addition to the focal herbivore (column *herbivores* in *studyData.csv*). Users can use this column to generate an inventory of biodiversity present on grazed and ungrazed sites.

We included the geospatial coordinates associated with the site or study whenever possible. These coordinates can be used with remotely sensed data and climate models. There is a database available^24^ that includes a global distribution of domesticated animals, which could be paired with the data provided in this paper. There is also an atlas of rangeland data that contains information about the distribution of rangeland types, key biodiversity areas in rangelands, and the number of threatened vertebrates in rangelands (https://www.rangelandsdata.org/). Climate datasets could be used to determine if the effects of grazing on biodiversity are a function of temperature or precipitation patterns. In addition to the year the study was published, we include the year the study was conducted, which could be used to determine the specific annual climate conditions. We recommend using the Climatic Research Unit (https://sites.uea.ac.uk/cru/) to determine daily resolved climate patterns for the last century^25^. For users interested in predicting future patterns of climate change under different global circulation models and shared socio-economic pathways (SSPs), we recommend using WorldClim^26^. For users interested in examining the consequences of grazing proximity to freshwater, the HydroLAKES database would be useful because it provides the locations for approximately 1.4 million lakes worldwide^27^. Finally, including data on annual plant productivity, such as estimates of above and below ground carbon density^28^, could effectively estimate grazing intensity among studies within our database. As exemplified above, these databases can complement the database presented here to answer ecological and sociological questions concerning sustainable grazing practices.

## Data Availability

We provide code in R that will assist in reviewing the database and examining patterns (https://afilazzola.github.io/GrazingDatabase/). We conducted data synthesis, technical validation, and visual quality assurance in R version 3.5.1 using *tidyr*, *dplyr*, *ggplot*^29^, and *raster*^30^ packages. Within the repository, we provide code for separating nested observations within the same cell, such as the list of herbivores reported in each study that are currently separately by semicolons. We also provide code for joining each meta-data file to the master file. All code used in the technical validation of this study is provided in the repository. All the code is written in R, except the code used for the consolidation step in technical validation, which is written in JSON. All code is freely available under the Massachusetts Institute of Technology license.
